# Editor’s Book Table

**Published:** 1870-05

**Authors:** 


### Editor's Book Table.

[Note.—All works reviewed in the columns of the Chicago Medical Journal may be found in the extensive stock of W. B. Keen & Cooke, whose catalogue of medical books will be sent to any address upon request.]

*A Practical Treatise on the Diseases of Children.* By J. Forsyth Meigs, M. D., etc., etc., and William Pepper, M. D., etc., etc. Fourth edition (of Meigs on Diseases of Children). Revised and greatly enlarged. Philadelphia: Lindsay & Blakiston. 1870. Pp. 921. $6.00.

From the Publisher's notice we extract:

"Dr. Meigs' work has been out of print for some years. The rapid sale of the three previous editions, and the great demand for a new edition, is sufficient evidence of its great popularity; while the very large practice of many years' standing of the author in the specialty of "Diseases of Children," imparts to it a value unequaled, probably, by any other work on the same subject now before the Profession. This present edition has been almost entirely rewritten and rearranged, and no effort or labor has been spared by either Drs. Meigs or Pepper, to make it represent fully in its most advanced state the present condition of Medicine as applied to Children's Diseases.

"The entire work has been subjected to careful revision. Several of the articles, as those on Eclampsia, Chorea, and Parasitic Skin Diseases, have been much enlarged; and others, as the various articles on the Diseases of the Stomach and Intestines, and that on Eczematous Affections, entirely rewritten. In addition, articles have been added upon the following important subjects:

| | |
|---|---|
| Diseases of the Heart. | Facial Paralysis. |
| Cyanosis. | Rheumatism. |
| Diseases of the Cæcum and Appendix. | Diphtheria. |
| Intussusception. | Mumps. |
| Chronic Hydrocephalus. | Rickets. |
| Tetanus Nascentium. | Tuberculosis. |
| Atrophic Infantile Paralysis. | Infantile Syphilis. |
| Progressive Paralysis, with apparent Hypertrophy of the Muscles. | Typhoid Fever. |
| | Sclerema. |

"The new matter thus added amounts to nearly 200 pages. It has been the effort of the authors, while endeavoring to make the work fully represent the state of our knowledge upon the subjects treated of, to retain its eminently practical character; and with this view, an unusually large amount of space has been devoted to the consideration of the treatment of each disease."

It will be seen from the above that the present is not a mere reprint of the previous work, or with more or less numerous additions. Those who have either of those editions need not be told that they are full of interesting matter; and we are pleased to see that in this edition improvement is everywhere visible. It is, in fact, so nearly a new treatise, that the possession of any other edition should not prevent purchase of this. We could select chapters, or even take them at random, worth more to the practitioner than what is charged for the work as a whole. It may rank among the most complete of the systematic treatises on the subject. The index is copious, and a glance at it will show the great range of topics considered.

*A Practical Guide to the Study of Diseases of the Eye; their Medical and Surgical Treatment.* By HENRY W. WILLIAMS, A. M., M. D., Ophthalmic Surgeon to the City Hospital, Boston; etc., etc. Third Edition, Revised and Enlarged. Boston: Fields, Osgood & Co. Philadelphia: Lindsay & Blakiston. 1869. Pp. 422.

To the active practitioner, who has not time to wade through the diffuse descriptions, hair-splitting divisions, and innumerable technicalities which befog everybody except *the* specialists themselves, in the more elaborate treatises on Diseases of the Eye, Dr. Williams' book will prove a welcome relief. It is concise, yet clear and practical. The first chapter discusses the modes of examination of the eye; the second, the Ophthalmoscope; and the third, remedies and their application. Then follow twenty-one chapters on Special Diseases of the organ and its appendages. The Appendix, profusely illustrated, gives a resume of recent advances in Ophthalmic Science. There is a full index, and a number of leaves giving test types, similar to those of Prof. Snellen, of Utrecht.

*Manual of Chemical Examination of the Urine in Disease; with brief Directions for the Examination of the most Common Varieties of Urinary Calculi.* By AUSTIN FLINT, Jr., M. D., Professor of Physiology and Microscopy in the Bellevue Hospital Medical College, etc., etc. New York: D. Appleton & Company, 90, 92 and 94 Grand Street. 1870. Pp. 82.

The object of this Manual is to furnish facts in convenient shape for the physician, whilst engaged in such urinary examinations as are now daily required of him. The author informs us that he commenced writing it merely to accompany a set of tests, etc., which Messrs. Tieman & Co., the eminent instrument makers, were arranging for the use of physicians, but it insensibly expanded to its present dimensions. It cannot be expected to take the place of the elaborate treatises, but is well adapted for the purpose set forth in the title page. A series of tables are given, which add much to its convenience and adaptation to use.

*A Practical Treatise on the Diagnosis, Pathology and Treatment of Diseases of the Heart.* By AUSTIN FLINT, M. D., Professor of the Principles and Practice of Medicine and of Clinical Medicine, in the Bellevue Hospital Medical College, etc. Second Edition; Thoroughly Revised and Enlarged. Philadelphia: Henry C. Lea. 1870. Pp. 550.

On the appearance of the first edition of this work, we cordially recommended it to the profession as "full, accurate and judicious." It is not too little to say, that "it stands at the head of all treatises on the subject." It is a standard text book in all the colleges, and is everywhere endorsed by the profession. The second edition has been carefully revised, many additions and alterations made, and much has been rewritten. The author states that the basis of the revision has been an analysis of about 450 cases recorded by him since the publication of the first edition. Without remembering any other of the valuable contributions of Prof. Flint to the literature of the profession, this book alone would rank him among its master minds and chief benefactors.

*A Hand-Book of Operative Surgery.* By JOHN H. PACKARD, M. D., One of the Surgeons of the Episcopal Hospital, Author of a Manual of Minor Surgery, etc., etc.; With 54 Steel Plates and Numerous Illustrations on Wood. Philadelphia: J. B. Lippincott & Co. 1870. Pp. 211.

It was the object of the author to illustrate, by at least one good method, every surgical operation in general use at the present day. In numerous instances, optional methods are given. It is strictly a manual of operations, no reference being made to diagnosis or treatment, except so far as they should influence the adoption or choice of operative measures. Directions and illustrations are based on the experience of the author in hospital and private practice, together with observations on the operations of others, and further correctness assured by careful comparison with standard works. Every form of instrument required by the surgeon is described and shown by cuts. As a whole, the book fills a place previously unoccupied. A vast amount of information is concisely and perspicuously set forth in the text, and the steel engravings (by Illman & Son,) are excellently well adapted for their purpose. The book will be found very convenient even by those who have been in long practice, and are in possession of the larger systematic treatises. To the younger surgeons and students it is invaluable. We most heartily recommend it to our readers. It is just the thing to glance over at the emergency so often occurring to the surgeon.

*The Preventive Obstacle, or Conjugal Onanism; the dangers and Inconveniences to the Individual, to the Family, and to Society, of Frauds in the Accomplishment of the Generative Function.* By L. F. E. BERGERET, Physician-in-Chief of the Arbois Hospital (Jura); translated from the third French Edition, by P. DEMARMON, M.D., 1 vol. 12mo., cloth, $1.50. Mailed on receipt of price; TURNER & MIGNARD, publishers, 109 Nassau street, N. Y.

With regard to this book the publishers remark:

"This Work, which has reached a third edition within a year in France, treats of a subject hitherto neglected by medical writers; but of the highest importance, whether from a medical or from a social point of view. Not only every physician, but every one interested in social science, should read attentively the facts and conclusions set forth by the author as the results of an exceptionally wide experience."

Aside from a certain tendency to exaggeration, and the attributing to *frauds*, what in many of the cases cited is clearly attributable to excess and other causes, this monograph is certainly worthy of perusal, and may afford valuable suggestions to physicians. We should say, "It is of the French, Frenchy," if Dr. Storer's "*Why Not?*" and "*Is it I?*" did not remind us that these things are getting naturalized in Yankee land.

### Pamphlets.

*On the "Sedative" Action of Calomel in Disease.* By FREDERICK D. LENTE, M. D., Cold Spring, N. Y. Read before the Duchess Co. Medical Society, N. Y., Jan 12, 1870. Pp. 24.

This is an attempt to induce practitioners to seek the well-known "sedative" action of Calomel, when given in scruple and half-dram doses, or even *plus* this. The double-action, stimulant, (energizer of tissue metamorphosis,) and sedative, (diminisher of the same,) Calomel enjoys in common with a multitude of other remedial agents. It is an action that has been often sought with success, just as blood-letting, prostration by tartar emetic, and narcosis by opium have often proved successful, or at least have been followed by recovery from disease. Dr. Lente gives his experience in the use of Calomel, in sedative doses, in epidemic dysentery, membranous croup, cholera morbus, and refers to its similar use by others in Asiatic cholera, pneumonia occurring in a tuberculous patient, &c., &c. He says that he is as much opposed to its indiscriminate use as an alterative, that is, in minute doses, with a view to mercurialization, as almost any one can be — doubts its prophylactic power in primary syphilis, and thinks that, with the powerful modern remedies which enable us to promptly act on the nervous system, and control the action of the heart, Calomel, as an alterative at least, is unnecessary in all forms of fever, in most diseases of the liver and alimentary canal, and also in peritonitis, iritis, pericarditis, and allied affections. In his view, the abuse it has received from those who have employed it or vituperated it, should not prevent us from using it in certain diseases of a more than ordinarily dangerous character, where other agents are ordinarily futile. Dr. Lente is especially enthusiastic in his commendation of sedative doses of Calomel in membranous croup, and adduces several unmistakable cases which recovered after taking repeated doses, varying from twenty grains to a teaspoonful each. "In fact," he says, "it is well to bear in mind, when using the 'Calomel treatment,' that there is more danger in giving too little than too much, or, (to speak definitely,) in giving less than twenty grains than a little over thirty." Half an ounce has been thus given, to a delicate child, in three or four days, and perfect recovery followed. "There does not appear to be such a thing as a *poisonous* dose of Calomel." The writer attributes its beneficial influence, principally, to reflex action — something, perhaps, due to its lessening the plasticity of the blood, and something to its relieving hyperaemia of the mucous membrane.

We do not imagine that the practical application of the teachings of this monograph will widely prevail, and therefore we omit the earnest protest which otherwise we should deem necessary.

Such a practice is based on the baldest empiricism, and the evidences of its success are, in a scientific sense, no more worthy of confidence than the countless "cures" which have, from time to time, been attributed to agents and methods of the most antipodal character. The newspapers are full of such things, and even the Homœopathists rely upon their medical multiplication table.

*Proceedings of the Homœopathic Medical Society of Ohio.* Fifth Annual Session. 1869.

*Mineral Wealth of Missouri.* I.—Mines and Mining Education. II.— Coal and Iron. By. Prof. C. D. WILBER, Inspector of Mining Lands. Fifth Thousand.

*The Annals of Iowa.* Published Quarterly by the State Historical Society at Iowa City. January, 1870. Edited by the Corresponding Secretary. $1.00 a year. Address F. Lloyd, Iowa City, Iowa.

*Twelfth Annual Report of the Chicago Charitable Eye and Ear Infirmary.* Presented by the Surgeons for the year 1869.

Eight hundred and seventy-three cases were treated at this institution during the year 1869 — making an aggregate of 5,355 since its first opening, in 1858. From the report we extract:

Consulting Surgeons — PROF. J. W. FREER, M. D.; PROF. H. A. JOHNSON, M. D. Attending Surgeons — EDWARD L. HOLMES, M. D.; EDWIN POWELL, M. D.

This Institution is not an Asylum for the hopelessly blind or deaf, but an Infirmary for the treatment of the poor, suffering from curable diseases of the eye and ear.

Communities, therefore, should not be at the expense of sending cases, which are not curable, to the Infirmary. The Trustees have no funds to appropriate for the traveling expenses of patients on their return home. They can, however, furnish clothing for a limited number of patients.

The State of Illinois in 1867, and again in 1869, appropriated $10,000 for the support of poor patients at the Infirmary. The Governor of Minnesota has at his disposal a small sum for patients of this class at the Infirmary, receiving treatment for diseases of the eye contracted during the late war. The Trustees have also a limited sum for the support of indigent sailors and soldiers.

As the funds of the Institution are insufficient for the constant maintenance of a large number of patients, it is hoped that communities in the State of Illinois, from which patients are sent for treatment, will each contribute a small sum of money to meet a part of the cost of their support while at the Infirmary. This will enable the largest possible number of poor to receive the benefits of the fund appropriated by the Legislature. Patients not residing in Illinois, must pay for their board during the whole period of their treatment.

*Patients from Illinois are admitted for both gratuitous board and treatment, on furnishing written certificates of their indigent condition, either from their physicians or from a supervisor of the county or town where they reside.*

*Patients from Illinois* (*or other States when there are vacant rooms*), *are admitted for gratuitous treatment who bring similar certificates that they can pay for their board alone, but not for their treatment.*

For the cost of the annual repairs of the Infirmary building, furniture, bedding, and other incidental expenses, the Institution is dependent upon funds raised by private contributions.

During the past year, an extension to the Infirmary building has been constructed, at considerable expense, to provide additional accommodations and comfort for the increasing number of patients. The Infirmary is more than ever in need of aid from the public.

Donations of money, fuel, furniture, and provisions, are, therefore, most earnestly solicited in aid of this charity.

The Infirmary is located at 16 East Pearson Street, first street north of Chicago Avenue, near the corner of North State Street, in a healthy portion of the city, near the lake, and is provided with all the conveniences necessary for the comfort and welfare of its patients.

The Dispensary of the Infirmary is open daily from 2 to 2½ P. M. for such poor patients, not boarding at the Infirmary, as may require treatment.

*Intermarriage of Kindred. Consanguineous Marriages not Forbidden by Moral or Physiological Law. Evils of Connubial Alliances between Persons of Diverse Race. Annual Address before the Eclectic Medical Society of the State of New York, January,* 1870. By ALEXANDER WILDER, M. D., President of the Society. Pp. 32.

*On the Physical Basis of Life.* By T. H. HUXLEY, LL. D., F. R. S. University Series No. 1. New Haven, Conn.: Charles C. Chatfield. 1870. Price, 25 cents.

This is the first of a series of Educational and Scientific Lectures, Addresses and Essays, now in process of publication, at the office of the College Courant, (Yale,) New Haven. No. 2 (in press) is on the Correlation of Vital and Physical Forces, by Prof. Geo. F. Barker, M. D., of Yale College. Price, 25 cents.

The author of the present lecture adopts Protoplasm as the physical basis of life, whether animal or vegetable. This material, so far as form is concerned, is entirely homologous in plants and animals, and in many cases it is a mere matter of convention whether we call a given organism an animal or a plant. The gist of Huxley's doctrine is in this sentence: "If the properties of water may be properly said to result from the nature and disposition of its component molecules, I can find no intelligible ground for refusing to say that the properties of protoplasm result from the nature and disposition of its molecules." Regarding the charge of materialism urged against his views, he argues with an ingenuity which will command attention:

"If we find that the ascertainment of the order of nature is facilitated by using one terminology, or one set of symbols, rather than another, it is our clear duty to use the former, and no harm can accrue so long as we bear in mind that we are dealing merely with terms and symbols. In itself it is of little moment whether we express the phenomena of matter in terms of spirit, or the phenomena of spirit in terms of matter; matter may be regarded as a form of thought, thought may be regarded as a property of matter — each statement has a certain relative truth. But with reference to the progress of science, the materialistic terminology is every way to be preferred."

But we have not space for the quotations which are suggested upon every page. Those who are thinking on these great questions should carefully peruse this production as a whole.

### Non-Professional Exchanges.

*Harper's Magazine*, *Harper's Weekly*, *Harper's Bazar.*—Harper's Magazine is undoubtedly, as claimed, the best sustained work of the kind in the world. Apart from its illustrations, it contains from fifty to one hundred per cent. more matter than any similar periodical issued in the English language. It is peculiarly an evidence of the culture of the American people, not in the narrowed interpretation given to the word culture, in Boston, and exemplified in the very wet *Atlantic*, where minnows do much more abound than whales, but in the peculiar combination of "the racy monthly, the more philosophical quarterly, blended with the best feature of the daily journal." The *Weekly*—"A complete Pictorial History of the Times," and the *Bazar*, "A Repository of Fashion, Pleasure, and Instruction," are respectively the models which a host of publishers attempt to imitate, but none have yet equaled. Each of these periodicals is afforded, in consequence of their immense circulation, at the extraordinary low price of $4.00 per year; two of them at $7.00, or all of them for $10.00; address Harper & Brothers, New York.

Our publisher will furnish either or all of the Harper periodicals at club rates with the *Journal.*

*College Courant.*—Yale, New Haven, Connecticut. This is a weekly paper edited and published by CHAS. C. CHATFIELD, A.M., assisted in the Scientific Department by Prof. G. F. BARKER, M.D. It is a handsomely printed sixteen page quarto, and is afforded at $4.00 a year; single copies, ten cents. It is not merely, as the name might suggest, an organ or exponent of Yale College, but gives us the current news of collegiate life throughout the country. Condensed accounts of both literary and scientific progress, and elaborate papers from men of letters and distinction, render it a valuable means of intercommunication of thought and knowledge. On another page we have noticed a series of lectures now in progress of publication by the same publisher.

*The American Builder*, of this city, sustains admirably, the high reputation it has achieved in arts, architectural and general.

It is not only a paper for architects, but for all persons interested in building. Our friends who contemplate the erection of homes or their ornamentation or improvement, would get many valuable hints by subscribing for the *Builder*. But what calls for the present notice is the beautiful premium engraving, offered by the publisher to subscribers, for the present year. It is a splendid 24 by 32 inch fine line engraving on heavy plate paper, of Washington Irving. Drawing by Darley, engraving by Ritchie. It is really a magnificent work of Art. The plate is new and the picture cheap at thrice the price asked both for it and the *Builder*. Address, with $3.00, Charles D. Lakey, publisher, 113 and 115 Madison street, Chicago.

*The Arts—Devoted to Science and Arts.* April number, 1870, (Vol. 1, No. 2.) This is a new periodical, published monthly, in this city, by Jos. M. Hirsh & Co., Nos. 10 and 12 South Water street. Subscription price, $1.00 per year. From a glance at the table of contents, it will be seen that readers are invited to a pleasant *melange* of art and science. The present number contains a portrait and sketch of Thomas Graham, the eminent Scotch chemist. We wish *The Arts* success.

*The European Mail.*—A weekly summary of news for North America; usual contents:

"Accidents; Art and Science; Births; Marriages, and Deaths; Commercial Summary; Correspondence; Court; Criminal; Emigration; Foreign and Colonial; Gazette; General Summary; Imperial Parliament; Ireland; Latest Shipping; Legal; Literary; Market Reports; Medical; Mercantile; Military; Miscellaneous; Music and the Drama; Natural History; Naval; Obituary; Political; Prices Current; Scotland; Shipping and Freights; Special American Notes; Sporting; Stocks; and Shares; Wills and Bequests, etc., etc. Subscriptions, payable in advance, 17s. 4d. per annum, inclusive of postage. Address the Proprietors *European Mail*, Colonial Buildings, 44 A, Cannon Street, London, E. C. (Postage pre-paid.")

From several copies of this paper which we have received, we are enabled to recommend it to those taking interest in foreign affairs.

